# Body Mass Records of Zoo‐Managed Rhinoceros (*Ceratotherium simum, Diceros bicornis, Rhinoceros unicornis*) as Compared to Field Data of Free‐Ranging Specimens

**DOI:** 10.1002/zoo.70034

**Published:** 2025-10-14

**Authors:** Elisa Garand, Christiane Krauss, Anna Hauffe, Max Hahn‐Klimroth, Dennis W. H. Müller, Paul W. Dierkes, Marcus Clauss, João Pedro Meireles

**Affiliations:** ^1^ Clinic for Zoo Animals, Exotic Pets and Wildlife, Vetsuisse Faculty University of Zurich Zurich Switzerland; ^2^ Department of Bioscience Education and Zoo Biology Goethe‐University Frankfurt Frankfurt Germany; ^3^ Zoological Garden Halle (Saale) Halle (Saale) Germany

**Keywords:** body condition, diet, ex situ, growth, obesity, Perissodactyla, size, weight

## Abstract

The body mass of zoo animals may differ from those in wild populations due to the different environmental and dietary conditions being offered under human‐managed care. These differences in body mass may impact health at both individual and population levels. In the case of rhinoceroses, this is relevant because of the distinct feeding requirements of each species and their susceptibility to obesity when inappropriate diets are offered. Here we attempt a comparison between the adult body mass records of the global zoo populations of rhinoceroses (white *Ceratotherium simum*, black *Diceros bicornis*, and greater one‐horned *Rhinoceros unicornis*) and the available body mass records of free‐ranging specimens in the literature. Body mass data from free‐ranging specimens is surprisingly scarce for white and greater one‐horned rhinos. Most adult zoo rhinoceroses are within the body mass range described for wild populations except female white rhinoceroses, which are, on average, heavier than free‐ranging females. Also, contrary to what has been described for natural habitats, zoo rhinoceroses do not show evidence for seasonal fluctuations in body mass, most likely due to the consistent nutrient supply in zoos. While obesity might be present across female white rhinos, and exacerbate other underlying health issues at the individual level in other taxa, this particular data set does not provide evidence that obesity is a population‐level phenomenon in black or greater one‐horned rhinos. Our findings should not weaken the efforts towards improving zoo rhinoceros nutrition and body condition monitoring.

## Summary

- Original body mass data for free‐ranging white and greater one‐horned rhinos is scarce.
- Compared to free‐ranging conspecifics, female white rhinos in zoos are possibly overweight.
- For male white rhinos, black rhinos of both sexes, and female greater one‐horned rhinos, zoo weights do not differ systematically from free‐range data.

## Graphical abstract

Female white rhinos in zoos systematically exceed the body mass range for free‐ranging specimens; this is not the case in black rhinos.

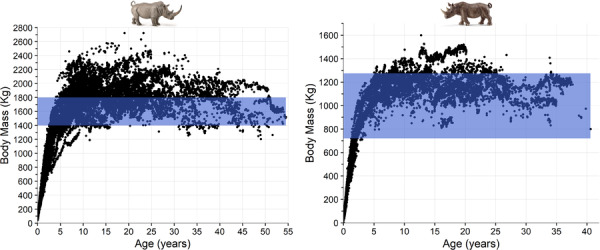

## 1 Introduction

Rhinoceroses (rhinos) have been managed in human care for centuries (Wittwer et al. 2023). The three species most commonly kept in zoos (white rhino *Ceratotherium simum*, black rhino *Diceros bicornis*, greater one‐horned [GOH] rhino *Rhinoceros unicornis*) differ in their natural diet and in their feeding requirements in human care (Clauss and Hatt 2006).

As monogastric herbivores with fermentation occurring in the hindgut, rhinos should be particularly susceptible to obesity, as any highly digestible diet component will be digested in their small intestine (Clauss and Dierenfeld 2008). This has been especially suggested for the GOH rhino (Atkinson et al. 2004; Clauss et al. 2005; Heidegger et al. 2016) and for the white rhino (Clauss and Hatt 2006). However, to our knowledge, large‐scale surveys on body mass or body condition in zoo rhinos are still lacking. By contrast, some authors have suggested that black rhinos might, also due to their more nervous nature, not be as susceptible to obesity (Clauss and Hatt 2006; Radeke‐Auer et al. 2023). On the other hand, however, obesity‐induced insulin resistance features prominently in a theory linking overfeeding of black rhinos to one of their most prominent health problems – iron overload disorder (Schook et al. 2015), and elevated body condition has been linked to reduced reproductive success in this species (Edwards et al. 2015).

To assess to what degree the body mass of zoo‐managed rhinoceroses deviates from that reported for their free‐ranging conspecifics, we compared data collected from the global zoo community via the Species360 database with published literature reports. In doing so, we also realized how poor the basis of data on free‐ranging animals actually is.

## 2 Methods

As part of Species360 research data use agreement # 84212, we received data on body masses of rhinoceroses recorded in the Zoo Information Management System (ZIMS) and stored by Species360 in January 2024 (Table 1). The data were provided to us anonymized, giving each individual animal a unique, random identifier, indicating only the animal's sex, the body mass entered by a zoo and the corresponding age of the animal and the date of weighing, but not the identity of the reporting zoo or any additional information on the animals status (e.g. pregnancy, lactation, disease). As a data set that originates from contributions by a very large number of persons, these raw data are prone to include implausible and faulty entries. The data set was provided with an indication of which data points were considered outliers by several automated correction procedures inspired by Garand et al. (2024). These included the automatic flagging of entries above 7000 kg, of outliers based on percentiles of a sliding window along the age for juveniles and adults, of outliers based on the residuals of generalized additive models for each individual trajectory with at least 7 measurements, and of outliers based on the residuals of a common generalized additive model for all growth trajectories. Based on visual judgment, these procedures removed the majority of outliers from the data set. However, some evident outliers still remained (e.g., if an outlier occurred in a sliding window with very few measurements and hence biologically implausibly wide percentiles), which were removed manually from the datasets. The cleaned data set is provided as an online supplement.

**Table 1:** Results of Gompertz model fit (according to $y=A\;{e^{-e}}^{-k\left(t-t_{0}\right)}$) to the age‐specific body mass data of females and males of three rhinoceros species kept in zoos, and the resulting threshold age for defining adulthood. *A* = asymptotic adult body mass; *k* = relative growth rate; *t*_*0*_ = time until maximum growth.

Species	Sex	Asymptote mass (*A*; kg)	Time to maximum growth (*t*_*0*_, years)	Relative growth rate (*k*; d^−1^)	Threshold age (years)
*Ceratotherium simum*	Female	1839 ± 2.3	2.19 ± 0.023	0.54 ± 0.005	6.9
	Male	2066 ± 2.7	2.17 ± 0.018	0.46 ± 0.004	8.1
*Diceros bicornis*	Female	1173 ± 1.3	2.07 ± 0.019	0.69 ± 0.005	5.4
	Male	1144 ± 1.7	2.65 ± 0.036	0.90 ± 0.010	4.4
*Rhinoceros unicornis*	Female	1793 ± 2.3	2.20 ± 0.020	0.46 ± 0.004	8.2
	Male	1939 ± 4.4	2.10 ± 0.026	0.48 ± 0.006	7.8

For each species, and for females and males separately, a Gompertz growth model was fitted to the data. This model yields an asymptotic weight which can be interpreted as the growth plateau as well as the time from birth till maximum growth rate (the latter was not used in this study) (Zullinger et al. 1984). Note that Gompertz models need not necessarily be the best models to fit growth data (reviewed in Veylit et al. 2021); here, we did not employ them to yield the most accurate data fit, but only to define the age at which animals typically reach adult size. The adequacy of the models was checked by inspecting the resulting model as graphed against the raw data (Figure S1 in the supporting material). We defined the age cut‐off from which data would be included in the calculation of an adult average as when 95% of this asymptotic mass was reached. The resulting parameter estimates and ages used as the cut‐off to define adulthood size (the threshold age) are given in Table 1.

Body mass was first averaged per individual (using only data above the adulthood cutoff), and then across the means of all individuals. After confirming the normal distribution of the sex‐specific body mass data of males and females using the Shapiro‐Wilk normality test, we tested for sexual dimorphism in the zoo population by an independent t‐test in case *p*(Shaprio–Wilk) > 0.05, and otherwise by a Mann‐Whitney‐U test, using python's scipy package.

Next, we calculated the average of all adult individuals weighed in a year, and tested the resulting averages for a pattern (increase or decrease) from the first year onwards for which data from at least 10 individuals were available. We did this for both body mass and age.

Additionally, two patterns were assessed visually by assessing the data for each individual animal separately: (i) regular, annual (i.e., seasonal) fluctuations in body mass, and (ii) a decrease in body mass towards later adult life. This process included a first pre‐selection based on the rules explained below, and a subsequent visual assessment of whether the data was deemed sufficient to evaluate the pattern, and whether the pattern was evident. This subjective approach was chosen because the purely mathematical approach we applied did not yield satisfactory results at visual verification. While we did not formally assess the reason for this discrepancy, we believe that the irregularity at which weighing events for individual animals are spaced makes a formally consistent approach very difficult.

For a pattern related to old age, only those individuals were pre‐selected for which at least three measurements in both, the first part of the expected lifetime as well as the second part of the expected lifetime (> 20–27.5 years, depending on species) were present. To assess seasonal fluctuations, pairs of measurements between one ‘winter’ and the subsequent ‘summer’ (or vice versa) were used. The data was divided into 6‐month periods corresponding to spring/summer and autumn/winter, and all measurements in these periods were averaged into a single data, and only these averages were plotted. The mathematical approach attempted assigned each pair a score (+1, 0, or −1) which signifies whether this corrected value was larger during summer, the same up to 0.5% between summer and winter, or larger during winter. The average over the scores per pair should yield an intuitive measure of a seasonality strength (−1 winter is larger than summer, 0 no difference, +1 summer is larger than winter). Setting arbitrary thresholds, a seasonality effect was suspected if this absolute score exceeded 0.3 and the score was built on at least six summer/winter pairs. However, in many cases the visual inspection of the plot did not corroborate the mathematical result. Therefore, the data of the pre‐selected individuals were plotted and all inspected individually, assessing subjectively whether the data was sufficient to evaluate the pattern, whether the pattern was visually evident (then the individual was counted) or not (then it was not). An example of such an individual plot is given in Figure S2. Using this information, we determined the proportion of individuals that showed a seasonally fluctuating body mass and a decline in body mass with progressing age late in adult life of all those in which we deemed the data sufficient to assess the pattern.

Data on body mass of free‐ranging rhinoceros were taken from the scientific literature (Table 2).

**Table 2:** Body mass records (in kg) for adult, free‐ranging rhinoceros species in natural habitats.

Source	*n*	Minimum	Average	Maximum
*Ceratotherium simum*				
		**Females**		
(Hillman‐Smith et al. (1986))°	6	1500	1600	1700
(Owen‐Smith (1988))°	—	1400*	1600	1800
(Fuentes‐Allende et al. (2023))	2	1530	1564	1598
		**Males**		
(Hillman‐Smith et al. (1986))	1	—	2130	—
(Owen‐Smith (1988))°	—	2000*	2200	2400
		***Unknown sex***		
(Mentis (1970))°	—		1724	
*Diceros bicornis*				
		**Females**		
(Meinertzhagen (1938))	5	997	1080	1276
(Wilson (1968))	2	862.3	925.1	987.9
(Hitchins (1968))	6	719.4	886.0	1134
		**males**		
(Meinertzhagen (1938))	10	1072	1159	1314
(Denney (1969))	4	1033	1125	1196
(Wilson (1968))	1	—	903.56	—
(Hitchins (1968))	8	709.0	853.9	1022.4
		**Unknown sex**		
(Mentis (1970))°	—		816	
*Rhinoceros unicornis*				
		**Females**		
(Atkinson (2002))°	4	1600	1825	2000
		**Unknown sex**		
(Eisenberg and Seidensticker (1976))°	—	1100	1203.33	1400
(Dinerstein (1989))°	—	1410	1705	2000

* Based on the range between the average and the maximum °estimates/undefined.

## 3 Results

Data on the body mass of free‐ranging rhinos are rare (Table 2). Sexual dimorphism has been suggested for the white rhino and the GOH rhino (Laurie 1982), but this is based on free‐ranging animals only in the case of the white rhino. For white rhinos, the source of body mass information (Owen‐Smith 1988) is mainly based on estimates with only a few actual measurements reported for a male (Hillman‐Smith et al. 1986) and two females (Fuentes‐Allende et al. 2023). For GOH rhinos, no reports of actual weighings seem to exist; information from different sources (Laurie 1982; Laurie et al. 1983) are typically derived from zoo speciemens (Lang 1961; Lang 1967). The data listed in Table 2 derives from estimates of free‐ranging females, and data without specification on method or sex for free‐ranging animals. Dinerstein (1991) states that the distinct sexual dimorphism described in the zoo literature (Lang 1961) does not correspond to his personal impressions in the wild, and that especially males may show higher growth rates in zoos. For black rhinos, more actual weighing data exists (Table 2).

Zoo animal data published previously, based on post‐mortem results in adults, indicated an average body mass of 924 kg (560–1400 kg) for female and 966 kg (650–1200 kg) for male black rhinos (Radeke‐Auer et al. 2023) and 1759 kg (1200–2500 kg) for female and 1711 kg (1251–2041 kg) for male GOH rhinos (Wyss et al. 2012). Additionally, measurements collected by a survey on GOH rhinos indicated an average of 1711 kg (1197–2026 kg) for females and 2031 kg (1441–2500 kg) for males (Heidegger et al. 2016).

**Figure 1:**
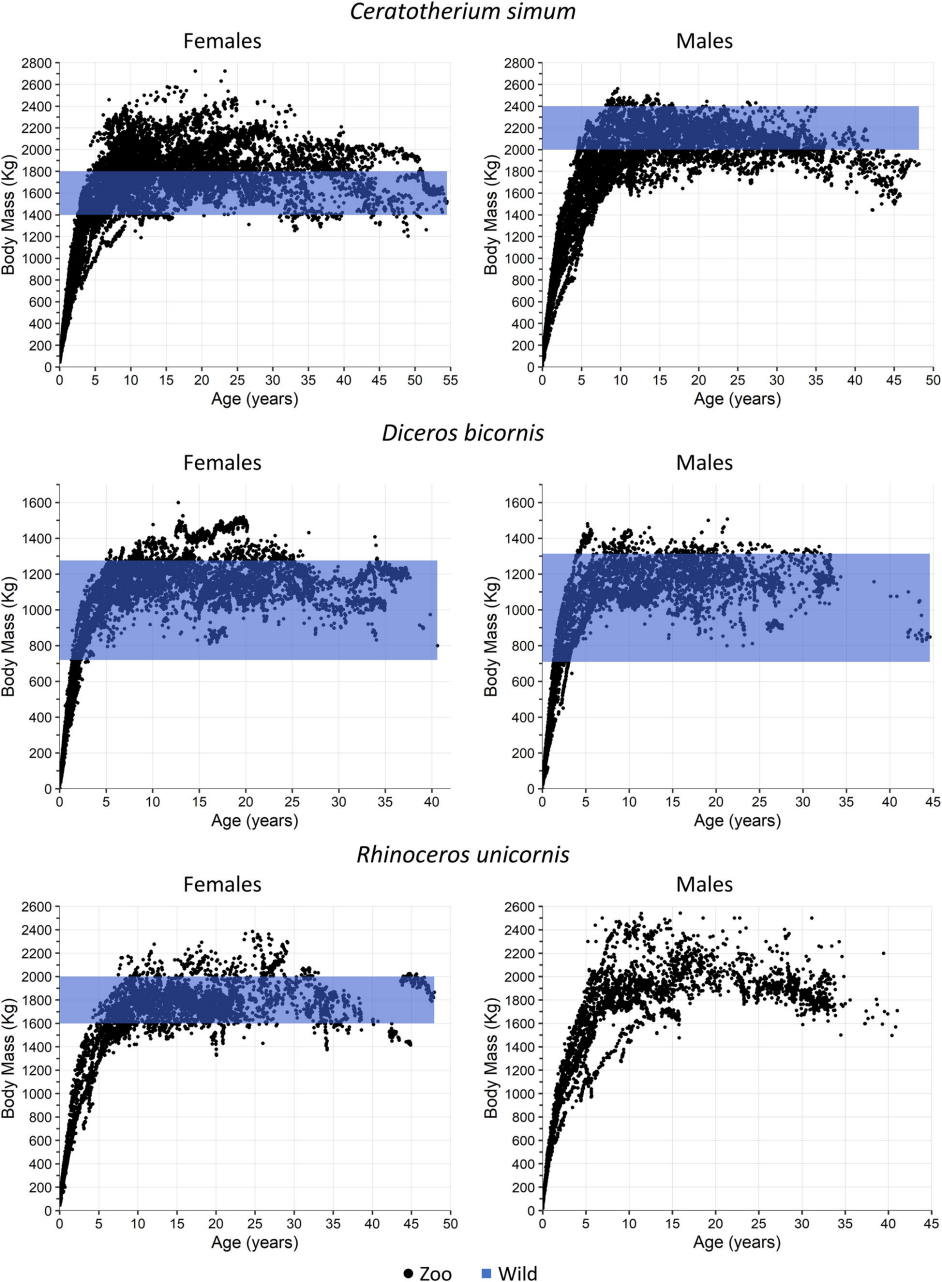
Body mass data for zoo‐kept rhinoceros species (black dots) as compared to the literature data range of adult, free‐ranging specimens (blue bands) (for sources, see Table 2). Note that this selection of individuals is based on data availability and not necessarily representative for the current global zoo populations.

The number of individuals and the descriptive statistics for the zoo data of the present study are given in Table 3. There were significant differences between females and males in all three rhino species (Table 3). GOH and white rhinos displayed the biggest sexual dimorphism in body mass, with males being 11% and 10% heavier on average than females, respectively. Male black rhinos are, on average, just 4% heavier than females. Compared to the sparse and often estimated data from the wild, female white rhinos in zoos are 226 kg or about 14% heavier on average. For male white rhinos, black rhinos of both sexes, and female GOH rhinos, the data yield no indication of a relevant discrepancy in adult body mass between natural habitats and zoos (Figure 1).

**Table 3:** Body mass records (in kg) and patterns for adult, zoo‐kept rhinoceros species. Note that this selection of individuals is based on data availability and not necessarily representative for the current global zoo populations. Percentages of individuals displaying seasonal fluctuations or old age decline expressed on the basis of all individuals in which the pattern could be assessed.

Sex	*n* individuals (total weighings)	Mean ± SD (min, max)	% seasonal fluctuations (of *n*)	% old age decline\(of *n*)
*Ceratotherium simum*				
Female	209 (10515)	1826 ± 201^A^ (1300, 2310)	18.5% (27)	66.6% (21)
Male	129 (6099)	2012 ± 188^B^ (1478, 2473)	19.0% (21)	76.9% (13)
*Diceros bicornis*				
Female	115 (7350)	1105 ± 169^a^ (560, 2050)	22.7% (22)	77.7% (18)
Male	117 (4035)	1149 ± 133^b^ (800, 1500)	11.1% (18)	71.4% (14)
*Rhinoceros unicornis*				
Female	54 (4861)	1805 ± 178^A^ (1470, 2270)	7.1% (14)	62.5% (8)
Male	55 (1994)	2007 ± 237^B^ (1354, 2542)	20.0% (10)	75.0% (8)

*Note:* AB, ab indicate significant sexual dimorphism, as assessed by parametric t‐test (A,B) or nonparametric U‐test (a,b) in the case of not normally distributed data.

**Figure 2:**
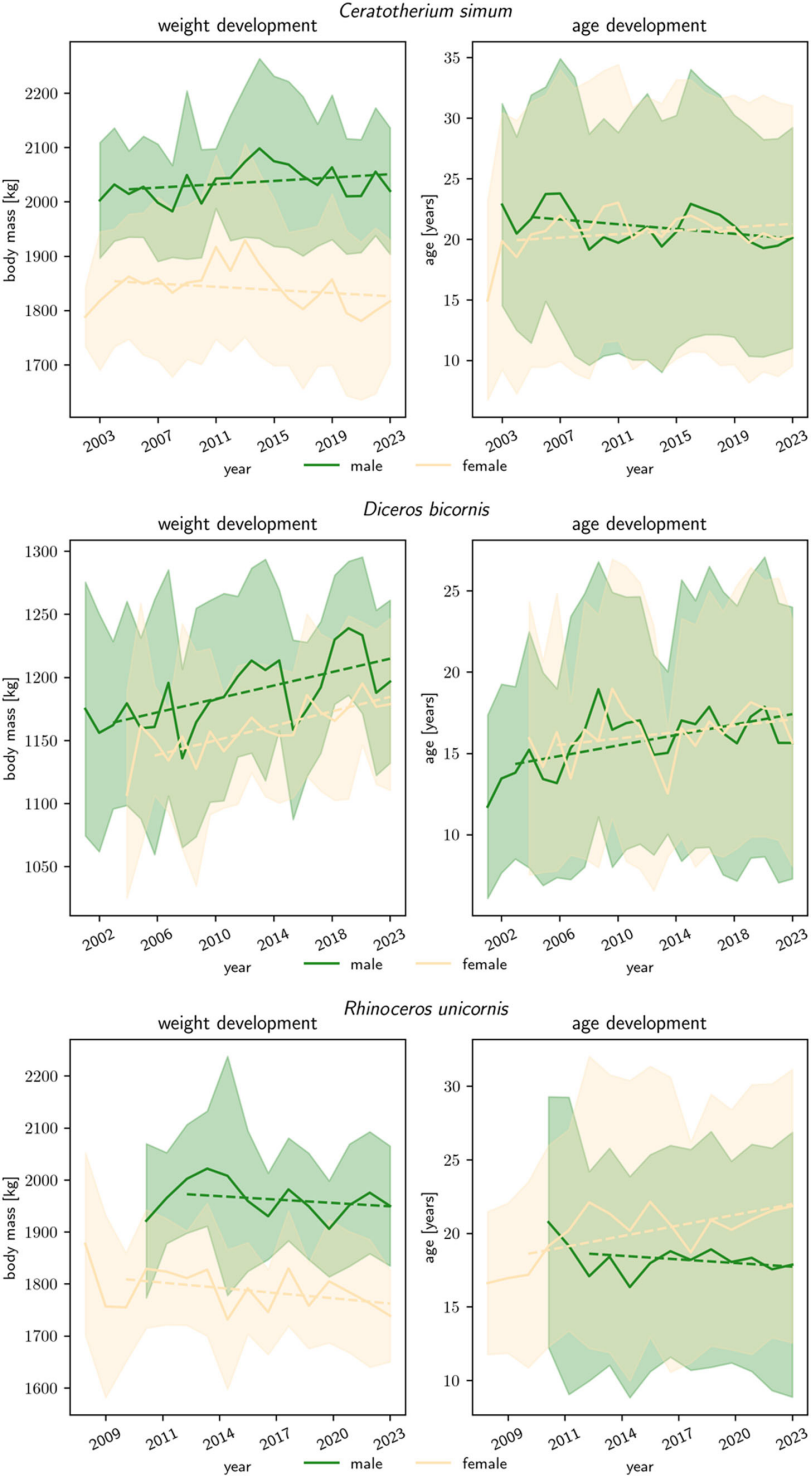
Historical trends (by year) in median (± quartile difference) body mass (left) and age (right) of adult, zoo‐kept rhinoceros. Means were used from the first year on where a minimum of 10 individuals were present in the data set. Note that this selection of individuals is based on data availability and not necessarily representative of the current global zoo populations. The number of individuals increased from 10 to 129 in female and 10 to 64 male white rhinos (*C. simum*), 20 to 34 in female and 14 to 33 male black rhinos (*D. bicornis*), and 11 to 36 female and 11 to 24 male GOH rhinos (*R. unicornis*).

Inspecting the historical trends of documented body mass data (Figure 2), there was a consistent increase in body mass in black rhinos of both sexes (in parallel with an increase in age); in white and GOH rhinos, the corresponding trends are mild, suggesting little systematic change on average adult body mass and age over time.

Seasonal body mass fluctuations occurred in 7–23% of assessable individuals; in other words, there appears to be no systematic seasonal body mass fluctuation in rhinos (Table 3). By contrast, a decline in body mass towards the later stages of adult life was evident in 63‐78% of assessable individuals (Table 3).

## 4 Discussion

The current study suggests that in general, the body masses recorded in zoo rhinos correspond to what is considered their body mass range in the wild, with the potential exception of female white rhinos. The findings need to be interpreted with caution for several reasons. Even if body mass is similar between two animal groups, such as free‐ranging and zoo animals, this could theoretically still mask differences in body condition where free‐ranging animals have more muscle mass and zoo animals more fat; more detailed investigations beyond sheer weighing would have to be performed to differentiate between different body compoments. Furthermore, the data used here are not necessarily representative of the zoo populations of the respective species, but of a group of zoos that have weighing facilities for their rhinos, and – possibly – also other aspects of advanced management. Nevertheless, given that for other zoo animals, such as certain primates (Leigh 1994; Pontzer 2023) or anteaters (*Myrmecophaga tridactyla*) (Garand et al. 2024), systematic deviations in the body mass of zoo animals compared to free‐ranging specimens have been reported, the present study can contribute to the discussion of overprovisioning of rhinos in zoos.

As outlined in the Introduction, the rhino species for which concern of overweight has most often been raised is the GOH rhino (Atkinson et al. 2004; Clauss et al. 2005; Clauss and Hatt 2006; Wyss et al. 2012; Heidegger et al. 2016). Body mass management is important in this species with respect to one of its most common health problems in zoos, foot sole lesions (Von Houwald and Flach 1998; Atkinson et al. 2004; von Houwald 2016). Even though a soft substrate is considered the single most crucial factor in preventing this problem (von Houwald 2016), maintaining body mass within species‐appropriate limits will help alleviate it. The slight downward trend in GOH rhino body mass in recent years (Figure 2), if indeed representative for zoo animals, should contribute to improved foot health in the species.

Based on subjective impressions, Clauss and Hatt (2006) suggested that white rhinos might be more prone to overweight in zoos than black rhinos – an impression supported by the present findings. Obesity‐related health problems have not been systematically reported in white rhinos, however; for example, an epidemiologic study on white rhino body mass or body condition across zoos is lacking so far to our knowledge. Therefore, our results should incite zoo managers to carefully evaluate their feeding regimes, in particular for white rhinos. Currently, the diets for white rhinos fed in Europe basically follow recommendations in terms of ingredient composition, mainly consisting of grass hay as considered adequate for this grazer species (Sauspeter et al. 2025). Therefore, if obesity is a concern, rather than considering a distinct diet change, choosing grass hay of a lower nutritional quality than the one currently fed appears as the most sensible option in this region.

For black rhinos, Schook et al. (2015) and Edwards et al. (2015) outlined scenarios that link the species’ particular susceptibility to iron overload disorder (IOD) and low reproductive success to obesity. Schook et al. (2015) based their interpretation on elevated insulin levels in zoo black rhinos, a finding previously suggested by Nielsen et al. (2012). The authors link the occurrence of IOD to an obesity‐induced, reduced insulin sensitivity in black rhinos. However, other systematic indicators of obesity in black rhinos in zoos are lacking, such as visual (body condition score), physical (e.g. ultrasound‐based subcutaneous fat thickness) or biochemical (e.g., serum leptin) measures (Abo el‐Maaty et al. 2017; Counotte et al. 2022). Of these, attempts to establish a viable serum leptin assay for the black rhino have failed so far (Schook et al. 2015). The present study also provides no indication of a systematic deviation in body mass from a natural state that could be linked to the systematic occurrence of IOD in the species (Paglia and Tsu 2012; Radeke‐Auer et al. 2023). Feeding regimes for black rhinos in European zoos, while potentially not ideal, also do not give rise to the suspicion of overfeeding (Sauspeter et al. 2025). Possibly, other causes of IOD in black rhinos than an obesity‐induced etiopathology should also be considered. Contrary to the other rhino species, our results show that in the last decade, black rhinos in zoos have a trend of increasing body mass (Figure 2) while still within the range of the specimens from natural habitats. At present, we cannot decide whether this should be interpreted as an increasing future risk for obesity, or as a sign that husbandry conditions are improving. Black rhinos are strict browsers (Clauss and Hatt 2006); browsers typically are considered challenging to feed in zoos (Clauss and Dierenfeld 2008; Radeke‐Auer et al. 2023). Ruminant browsers kept in zoos such as giraffes (*Giraffa camelopardalis*) (reviewed in Clavadetscher et al. 2021), moose (*Alces alces*) (Shochat et al. 1997; Clauss et al. 2002) and others (Wright et al. 2011; Taylor et al. 2013; Gattiker et al. 2014) have often been reported as having poor body conditions, interpreted as a consequence of a lack of browse forage provision and the ingestion of inappropriate concentrate feeds, which disrupt proper rumen function. By contrast, hindgut fermenting browsers or mixed feeders should not be similarly prone to a poor body condition triggered by a low‐forage, high‐energy diet, as exemplified by zoo‐managed tapirs (*Tapirus* spp.) (Pérez‐Flores et al. 2016) or elephants (Schiffmann et al. 2020). It is recommended that the body mass development in black rhinos is closely monitored to detect any possible shift towards obesity.

In our data, there was no indication for systematic seasonal fluctuations in body mass in any of the three rhino species, most certainly resulting from the constant supply of food being offered in zoos. The natural habitat of African rhinos suffers remarkable fluctuations in food and water availability and the body condition or mass of large herbivores in these seasonal habitats are impacted by seasonal variation of resources (Marshal et al. 2012; Fuentes‐Allende et al. 2023). It has been suggested that white rhinos cope with these fluctuations by using their body reserves during the dry season (Shrader et al. 2006), leading most certainly to fluctuations in body mass (and condition) during the year. This strategy has been suggested to allow them withstanding longer periods of food scarcity without having to increase foraging time or the intake of lower quality food (Shrader et al. 2006). Similar seasonal fluctuations have been suggested for black and GOH rhinos, too (Hrabar and Toit 2005; Medhi and Saikia 2020). However, rhinos are basically nonseasonal breeders (Radeke‐Auer et al. 2022), which suggests that seasonal body mass fluctuations may not be necessary to comply with their species‐specific physiology. This contrasts with species for which regular, seasonal changes in body mass and food intake are evident even under conditions of human care, such as deer (e.g., Schwartz et al. 1987; Armeni et al. 2022), bears (Hashimoto and Yasutake 1999; Gerstner et al. 2016), pinnipeds (Rosen et al. 2021) or cetaceans (Scala et al. 2025).

Although all species display statistically significant sexual dimorphism in body mass in zoos (Table 3), the much smaller dimorphism in black rhinos seems to corroborate the descriptions that free‐ranging black rhinos show no difference in body mass (Freeman and King 1969), while white rhinos have a marked difference between the sexes (Owen‐Smith 1988). Dinerstein (1991) states that zoo GOH rhinos show a much bigger sexual dimorphism than what he observed in wild populations. Possibly, the high food availability in zoos allows males to grow to their full potential; until comparative data from free‐ranging specimens become available, this hypothesis remains untested.

In conclusion, while the findings of the present study should not lead to a reduction of attention given to the monitoring of body condition in zoo rhinos, the data set does not indicate that obesity, while surely occurring in individual animals, is a population‐wide phenomenon in zoo rhinos, with the possible exception of female white rhinos. To gain a better overview of the body condition and nutritional status of zoo rhinos and their development over time, regular surveys using a body condition score (Reuter and Adcock 1998; Versteege and van den Houten 2011; Heidegger et al. 2016) would be welcome. With the ease that rhinos can be trained for medical procedures (Holden et al. 2006; Sullivan et al. 2020), supporting monitoring measures, for example based on serum biochemistry as suggested for horses (Abo el‐Maaty et al. 2017; Counotte et al. 2022), could also be established.

## Ethics Statement

The authors have nothing to report.

## Conflicts of Interest

The authors declare no conflict of interest.

## Supporting information

EGCKAH_RhinoBodyMassR1_Supplement_250622_mc.

## Data Availability

The data that supports the findings of this study are available in the supporting material of this article.
