# Supplementary material for: Body Mass Records of Zoo‐Managed Rhinoceros (Ceratotherium simum, Diceros bicornis, Rhinoceros unicornis) as Compared to Field Data of Free‐Ranging Specimens
Source: Zoo Biol. 2025 Oct 14;45(1):74–83. doi: 10.1002/zoo.70034 (PMC12884244; doi:10.1002/zoo.70034)
Supplement: Supplementary file 1 — EGCKAH_RhinoBodyMassR1_Supplement_250622_mc. [file ZOO-45-74-s001.docx]

***Supplementary material***

**Body mass records of zoo-managed rhinoceros (*Ceratotherium simum, Diceros bicornis, Rhinoceros unicornis*) as compared to field data of free-ranging specimens**

**Elisa Garand, Christiane Krauss, Anna Hauffe,** **Max Hahn-Klimroth, Dennis W. H. Müller, Paul W. Dierkes, Marcus Clauss, João Pedro Meireles**

| White rhino |  |
| --- | --- |
| 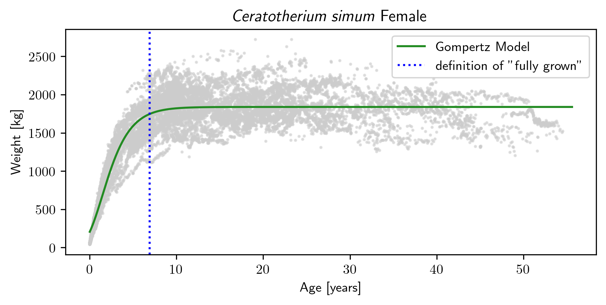 | 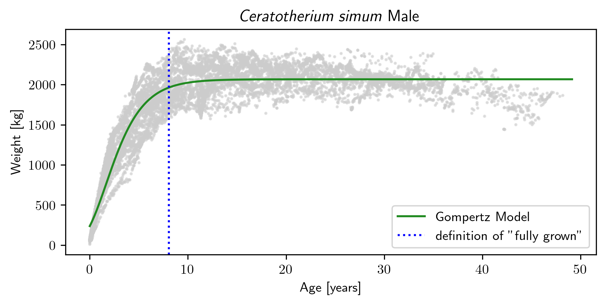 |
| Black rhino |  |
| 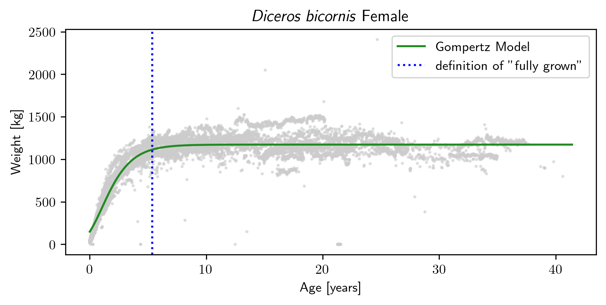 | 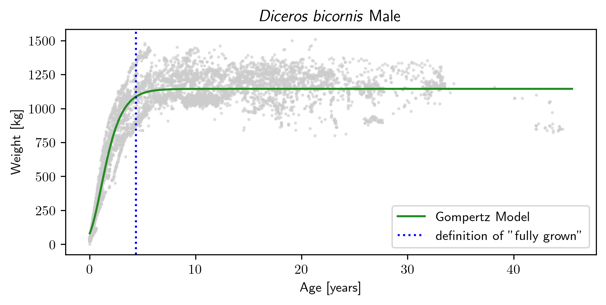 |
| Greater one-horned rhino |  |
| 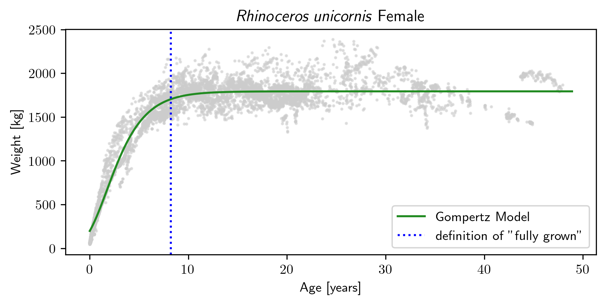 | 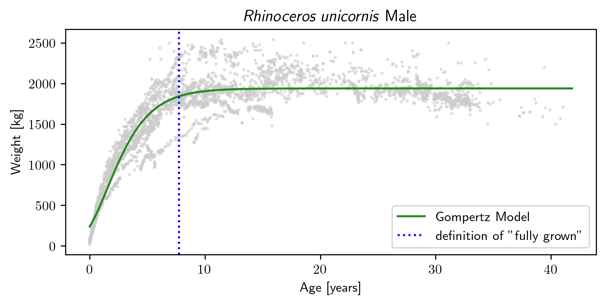 |

**Figure S1** Gompertz growth models (green line) fitted to the age-specific body mass data of females and males of three zoo-kept rhinoceros species. For a description of the model parameters, see Table 1.


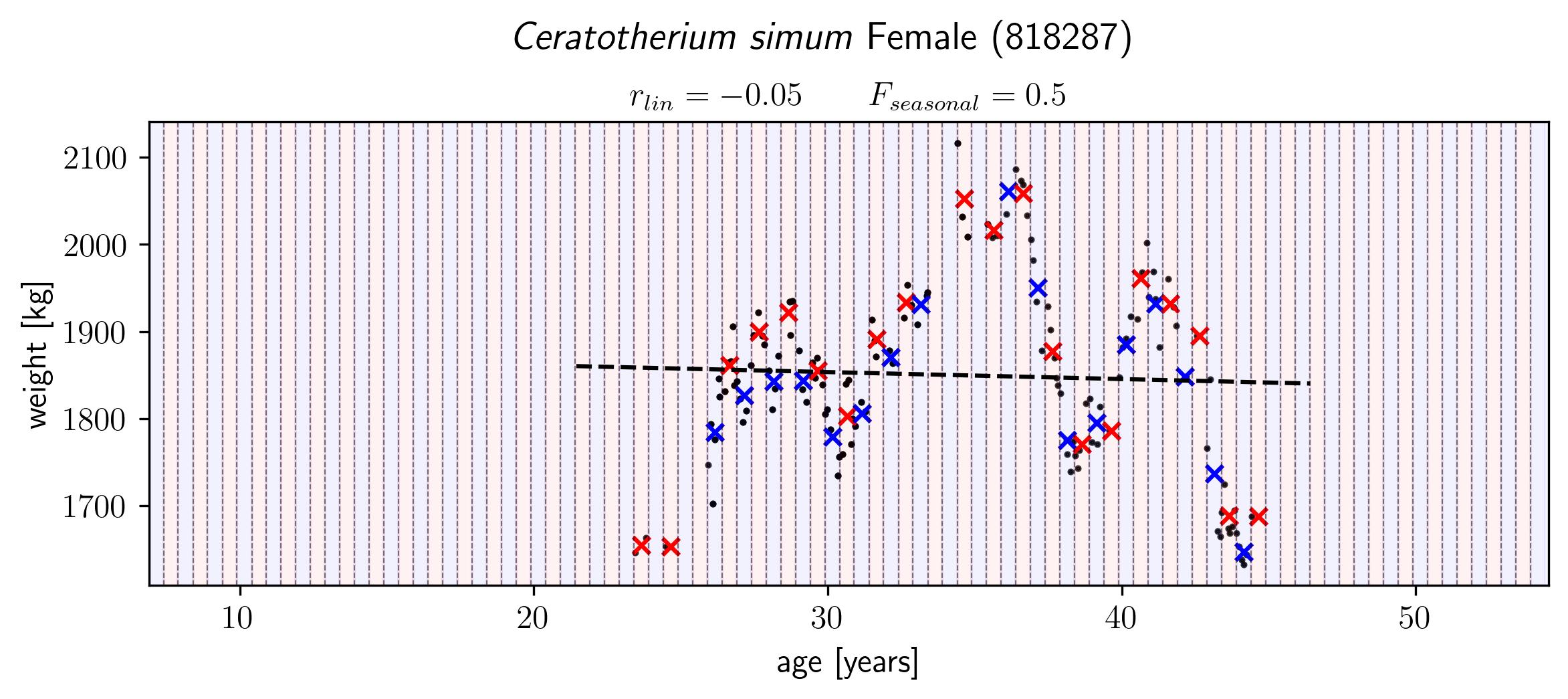


**Figure S2** Example for the visual display of body mass development in a female white rhinoceros. Dots represent original data, crosses the means for winter (blue) and summer (red) values. Note a general trend of body mass decline with age, an irregular change of body mass in cycles spanning several years, and several instances of a seasonal (annual) fluctuation.
